# Atomistic and Experimental Investigation of the Effect of Depth of Cut on Diamond Cutting of Cerium

**DOI:** 10.3390/mi9010026

**Published:** 2018-01-13

**Authors:** Junjie Zhang, Maobing Shuai, Haibing Zheng, Yao Li, Ming Jin, Tao Sun

**Affiliations:** 1 Center for Precision Engineering, Harbin Institute of Technology, Harbin 150001, China; zheng_hai_bing@126.com (H.Z.); jlsljm@hotmail.com (M.J.); 2 Science and Technology on Surface Physics and Chemistry Laboratory, Mianyang 621908, China; bk7041638@sina.com

**Keywords:** cerium, diamond cutting, depth of cut, phase transformation, molecular dynamics

## Abstract

The ultra-precision diamond cutting process exhibits strong size effects due to the ultra-small depth of cut that is comparable with the cutting edge radius. In the present work, we elucidate the underlying machining mechanisms of single crystal cerium under diamond cutting by means of molecular dynamics simulations, with an emphasis on the evaluation of the effect of depth of cut on the cutting process by using different depths of cut. Diamond cutting experiments of cerium with different depths of cut are also conducted. In particular for the smallest depth of cut of 0.2 nm, shallow cutting simulations varying the sharpness of the cutting edge demonstrate that an atomically sharp cutting edge leads to a smaller machining force and better machined surface quality than a blunt one. Simulation results indicate that dislocation slip is the dominant deformation mechanism of cerium under diamond cutting with each depth of cut. Furthermore, the analysis of the defect zone based on atomic radial distribution functions demonstrates that there are trivial phase transformations from γ-Ce to δ-Ce occurred in both the machined surface and the formed chip. It is found that there is a transition of material removal mode from plowing to cutting with the increase of the depth of cut, which is also consistent with the diamond cutting experiments of cerium with different depths of cut.

## 1. Introduction

An ultra-smooth surface is of significant importance for achieving high performance of parts and components that are used in fields such as precision instruments, aerospace and nuclear energy, etc. For instance, surface roughness has a significant impact on both the corrosion and oxidation resistances of metallic surfaces, which subsequently influence functionalities and life cycles of metal parts and components [1,2,3]. In particular for cerium, which is one of the most active lanthanide elements with high chemical activity, an ultra-smooth surface with nanometer surface roughness is greatly desired to improve its oxidation resistance. The diamond cutting technique has been demonstrated to be an important method for preparing ultra-smooth surfaces [4,5,6]. To facilitate the improvement of machined surface quality, a thorough understanding of machining mechanisms involved in the diamond cutting process is critical. In line with experimental study, theoretical investigation based on the molecular dynamics (MD) simulation has also been widely conducted to explore the ongoing diamond cutting process. 

Theoretical investigation and experimental studies both indicated that there is a strong depth of cut (DOC) size effect in the diamond cutting process, giving the ultra-small DOC that is comparable with the cutting edge radius. Lucca et al. found that the increase of the negative rake angle leads to a significant increase in the DOC for the onset of surface fracture in the orthogonal cutting of Ge [7]. Li et al. performed MD simulations of diamond tip grinding of copper, and found that a large DOC results in a large chipping volume and a great temperature rise in the copper workpiece, and a small DOC will reduce the subsurface damage and improve the smoothness of a ground surface [8]. Zhu et al. found that a large DOC results in a large material deformation and large chip volume, as well as high cutting force and normal force in the nanometric cutting of copper using diamond tools [9]. Pramanik et al. found that the low DOC leads to low machined surface quality in terms of surface roughness and surface appearance due to higher cutting tool wear [10]. 

Although valuable insights have been obtained in previous studies, the influence of DOC on the diamond cutting of cerium is largely unknown. Diamond cutting is a highly coupled process between the workpiece material and the cutting tool, which implies that the properties of the workpiece material have a strong impact on the machining process. It’s known that cerium has a rich path of high temperature and high pressure phase transformation [11,12,13]. In the diamond cutting process, there are zones with high hydrodynamic pressure formed in the interface between the cutting tool and the workpiece material. In addition, the friction between the rake face and the formed chip, as well as the friction between the clearance face and the machined surface, leads to high local temperatures. However, it is still largely unknown whether there is phase transformation in the diamond cutting of cerium. The changes in local crystal structures accompanied by phase transformation lead to changes in the mechanical properties of the workpiece material, which subsequently result in a strong nonuniform machined surface quality in the cutting process. Furthermore, cerium, as a metallic material, exhibits high dislocation-mediated plasticity [14]. Although it is known that dislocation slip plays a key role in the plastic deformation of face-centered cubic (FCC) metals in the cutting process, there are certain questions raised but not answered: what are the deformation modes of cerium under diamond cutting? Furthermore, what is the dependence of diamond cutting of cerium on DOC? 

Therefore, in the present work, we investigate the underlying machining mechanisms of cerium under diamond cutting. Specifically, we focus on microscopic deformation modes of cerium in terms of dislocation slip and phase transformation, and their correlation with macroscopic machining results in terms of machining force and machined surface morphology. We also explore the dependence of machining mechanisms on DOC by performing both MD simulations and experiments of diamond cutting of cerium with different DOCs. 

## 2. Simulation Method

**Figure 1:**
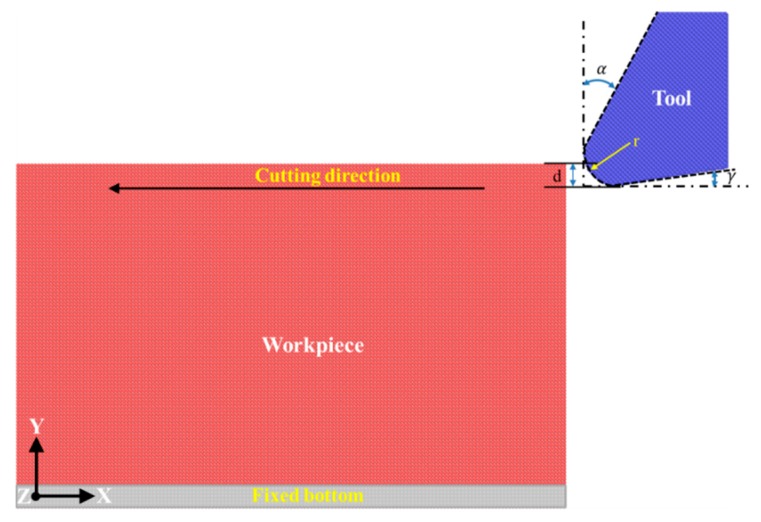
Molecular Dynamics (MD) model of diamond cutting of cerium.

Figure 1 presents the MD model of diamond cutting used, which consists of a single crystal cerium workpiece and a diamond cutting tool. The cerium workpiece is composed of γ-Ce in an FCC lattice structure. The workpiece has dimensions of 41, 25 and 31 nm in the *X*, *Y* and *Z* direction, respectively. Periodic boundary condition is only applied in the *Z* direction of the workpiece. Furthermore, the bottom of the workpiece is fixed to prevent any rigid motion during the cutting process. As illustrated in Figure 1, the diamond cutting tool has a cutting edge radius of 2 nm. Furthermore, the diamond cutting tool has a rake angle and clearance angle of 30° and 10°, respectively. The diamond cutting tool is treated as a rigid body which does not deform in the cutting process due to its ultra-high hardness, as compared to metallic cerium. The Ce–Ce interactions in the cerium workpiece are described by the embedded atom method (EAM) parameters for cerium developed by Sheng et al., which are capable of accurately describing bulk elastic properties of FCC cerium phases [15]. The Ce–Ce interactions between the cerium workpiece and the diamond cutting tool are described by the pairwise Morse potential. The detailed parameters for the utilized Morse potential can be found in recent work [16]. 

The MD simulation of diamond cutting consists of two stages, first equilibrium and second cutting. In the equilibrium stage, atoms in the cerium workpiece are relaxed first by energy minimization and then by NVT (constant atom number N, constant volume V and constant temperature T) dynamic relaxation at 3 K. After the equilibrium, the relaxed workpiece is subjected to diamond cutting using the diamond cutting tool in the NVT ensemble. The cutting process is terminated when the workpiece material displaced by the cutting tool is completely separated from the workpiece. Five DOC: 0.2 (d1), 0.6 (d2), 1.0 (d3), 1.5 (d4) and 2.0 (d5) nm, are considered to address the influence of DOC on the cutting process. For each DOC, the cutting speed is 100 m/s. Lattice defects generated in the cutting process are analyzed by the common neighbor analysis (CNA) [17]. Furthermore, different cerium phases are identified by using the radial distribution function (RDF). All the MD simulations are performed using the large-scale atomic/molecular massively parallel simulator (LAMMPS) code with an integration time step of 1.0 fs [18]. The OVITO is employed to visualize MD data and generate MD snapshots [19]. The details of the utilized MD model can also be found elsewhere [16].

## 3. Results and Discussion

### 3.1. Sharp vs. Blunt Cutting Edge

It has been demonstrated that the geometry of the diamond cutting tool has a prominent influence on the cutting process. Therefore, the influence of cutting edge sharpness on the diamond cutting of cerium is first investigated by using two types of cutting edge: an atomically sharp one with an edge radius of 0 nm and a blunt one with an edge radius of 2 nm. For each type of cutting edge, the rake angle and clearance angle are 30° and 10°, respectively. The DOC and cutting speed utilized in the two cutting processes are 0.2 nm and 100 m/s, respectively.

**Figure 2:**
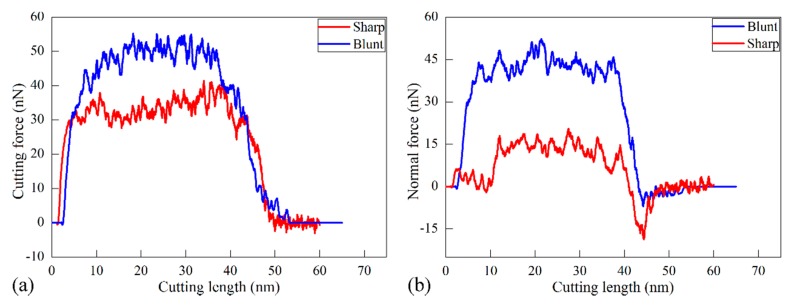
Variations of cutting force (**a**) and normal force (**b**) with cutting length during cutting processes with different types of cutting edge.

**Figure 3:**
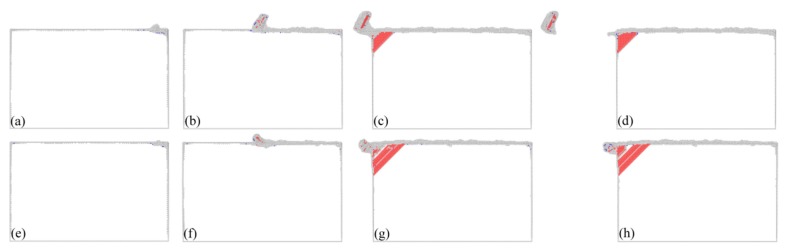
Cross-sectional view of instantaneous defect structures generated in the cutting process with a sharp (upper) and a blunt (bottom) cutting edge. Cutting distance: (**a**) and (**e**) 5 nm; (**b**) and (**f**) 23 nm; (**c**) and (**g**) 45 nm; (**d**) and (**h**) 60 nm.

There are three machining force components acting on the diamond cutting tool: the cutting force along the *X* direction, the normal force along the *Y* direction and the lateral force along the *Z* direction, respectively. Figure 2a,b plot variations of cutting forces and normal forces with cutting length in the two cutting processes, respectively. Simultaneously, Figure 3 presents instantaneous defect structures formed in the workpiece at different cutting lengths with different cutting edge sharpness, aiming to reveal the microscopic deformation behavior of workpiece materials. Atoms in Figure 3 are colored according to their CNA values, and perfect FCC atoms are not shown for clear visualization of defects.

It is seen from Figure 2 that variations of both cutting force and normal force for each type of cutting edge have similar characteristics. Both cutting force and normal force have negative values when the cutting tool is approaching the workpiece, due to the adhesion between the cutting tool and the workpiece. When the cutting tool begins to come into contact with workpiece, the machining forces increase rapidly. Meanwhile, Figure 3a,e show that there no defect is generated beneath the machined surface, indicating that the rapid increase of machining forces is accompanied by elastic deformation of the workpiece material. With the further advance of the diamond cutting tool, the machining forces drop due to the plasticity initiation in the workpiece material, as shown in Figure 3b,f. Then the machining forces fluctuate around stable values when the cutting process is stable in the cutting length ranging from 10 to 35 nm. Dynamic inspection of the defect evolution indicates that there are considerable dislocations generated beneath the machined surface. Specifically, successive events of dislocation nucleation and subsequent glide lead to oscillations of machining forces shown in Figure 2. Figure 3c,g also show that there are dislocations accumulated in the vicinity of the left fixed free surface. Furthermore, dislocations have the same geometry with respect to the machined surface for different types of cutting edge. Figure 3d,h show that dislocations are remained within the material after the diamond cutting tool is separating from the workpiece. Correspondingly, Figure 2 shows that the machining forces drop precipitously. 

Figure 2 and Figure 3 jointly show that the cutting edge sharpness has a significant influence on the cutting processes. Figure 2 indicates that the critical cutting length for the first drop of machining forces is 2.3 and 7.8 nm at for the sharp and the blunt cutting edge, respectively, indicating that plastic deformation takes place earlier in the cutting process with the sharp cutting edge. Figure 2 indicates that machining forces for the blunt cutting edge are significantly higher than those for the sharp cutting edge. Furthermore, although the cutting force is larger than the normal force for each type of cutting edge, the differential value between the two force components is significantly different. In particular for the stable cutting period, the ratio of cutting force to normal force is 1.1 and 2.4 for the blunt and sharp cutting edge, respectively. 

**Figure 4:**
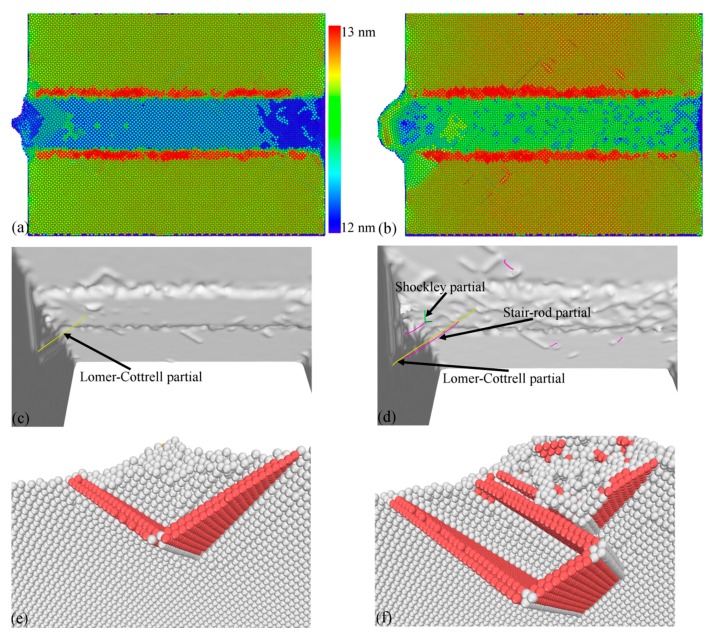
Machined surface morphologies (top row), defect characterization (middle row) and dislocation structure (bottom row) after cutting processes with two types of cutting edge. (**a**), (**c**) and (**e**): sharp cutting edge; (**b**), (**d**) and (**f**): blunt cutting edge. Atoms in (**a**) and (**b**) are colored according to their atomic heights. Atoms in (**e**) and (**f**) are colored according to their common neighbor analysis (CNA) values.

Figure 3 shows that a chip is formed for the sharp cutting edge, and there are considerable dislocations observed in the formed chip. In contrast, there is no chip formed for the blunt cutting edge. Figure 4a,b present machined surface morphologies after cutting processes with the sharp and blunt cutting edge, respectively. While the volume of surface pile up accumulating on both sides of the formed groove is significantly smaller for the sharp cutting edge than that for the blunt one, the integrity of the machined surface for the sharp cutting edge is also higher than that for the blunt cutting edge. These observations jointly demonstrate that a better machined surface quality can be achieved by using the cutting tool with a sharp cutting edge.

Figure 3 indicates that the cutting edge sharpness also has a prominent influence on the dislocation-dominated deformation of the workpiece material. Although the activated slip planes are similar for the two cutting processes due to the same crystallographic orientation, the dislocation slip event is more pronounced for the blunt cutting edge than that for the sharp cutting edge, indicating that a more serious plastic deformation occurred.

Figure 4c,d further characterize dislocation structures after the cutting process with the sharp and blunt cutting edge, respectively. Figure 4c shows that there is only one Lomer–Cottrell lock and it has a Burgers vector of 1/3[1 0 0]. Figure 4e further presents the atomic configuration of the Lomer–Cottrell lock, in which atoms are colored according to their CNA values. The Lomer–Cottrell lock is a sessile dislocation structure that locks the dislocation slip in two adjacent slip planes. There is also a Lomer–Cottrell lock formed in the cutting process for the blunt cutting edge. However, there are other two types of dislocations formed, i.e., stair-rod dislocation with a Burgers vector of 1/6[1 1 0] and a Shockley partial with a Burgers vector of 1/6[1 1 2]. Figure 4f further presents the atomic configurations of the dislocation structures, in which atoms are colored according to their CNA values. Furthermore, Figure 4d clearly shows the nonsymmetrical distribution of dislocation along the formed groove for the blunt cutting edge. The sharpness-dependent machining forces can be attributed to the different effective rake angles. For the blunt cutting edge with an edge radius of 2 nm, the effective rake angle is negative at the shallow DOC of 0.2 nm. In contrast, the effective rake angle is a constant value of 30° for the sharp cutting edge. 

**Figure 5:**
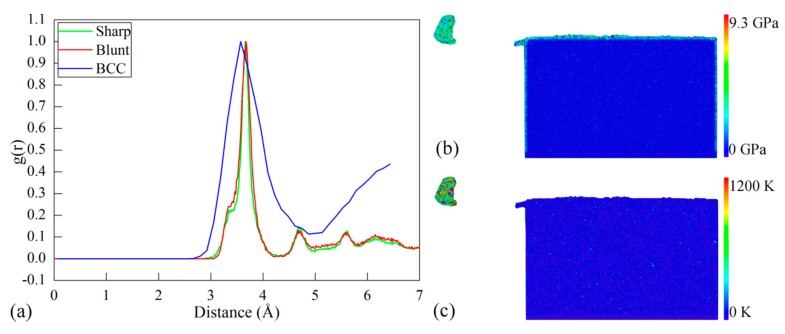
(**a**) Evolution of radial distribution function (RDF) in machined cerium workpice in the diamond cutting with different types of cutting edge. Contours of workpiece colored by (**b**) atomic stress and (**c**) temperature. BCC: body-centered cubic.

While the δ-Ce in the body-centered cubic (BCC) structure is stable at high temperature and low pressure, the γ→δ phase transformation may happen due to the high heat dissipation generated in the cutting process. In addition to dislocation slip, the propensity for phase transformation in the cutting processes with different types of cutting edge is also evaluated by examining the RDF evolution of the cerium workpiece. According to the CNA analysis of the machined workpiece, the ratio of other newly generated atoms except for FCC, hexagonal close-packed (HCP) and free surface atoms to the total atoms is 0.0004. The RDF analysis is performed on the defect zone that excludes FCC and HCP atoms, which is used to magnify the change of RDF peaks. Figure 5a plots the RDF of the workpiece after cutting processes with the sharp and blunt cutting edges. Furthermore, the RDF of the δ-Ce is also presented for comparison. It can be seen from Figure 5a that the sharp and blunt cutting edges yield similar RDF curves of the workpiece material. For each type of cutting edge, the first peak of the RDF curve of the machined surface closely coincides with that of the BCC cerium, indicating that there is phase transformation from γ-Ce to δ-Ce in the machined surface. Moreover, the RDF analysis on the formed chip also indicates that there is the same phase transformation from γ-Ce to δ-Ce in the chip. Figure 5b,c present contours of workpiece colored by atomic stress and temperature. It is seen from Figure 5b that although the highest stress approaches 9.3 GPa, the main part of the machined surface has a low stress of 2 GPa. Figure 5c indicates that the atoms with the highest temperature of 1000–1200 K are overwhelmingly located in the formed chip. The highest stress and temperature formed in the cutting process coincide with the stable condition of δ-Ce [11,16,20]. 

### 3.2. Influence of DOC

Diamond cutting simulations of single crystal cerium at different DOCs are conducted to address the influence of DOC on the cutting processes. For each DOC, the same diamond cutting tool with a blunt cutting edge radius of 2 nm is employed. Five DOCs: 0.2 (d1), 0.6 (d2), 1.0 (d3), 1.5 (d4) and 2.0 (d5) nm, are considered. Correspondingly, the ratio (*R*) of DOC (*d*) to cutting edge radius (*r*) is 0.1, 0.3, 0.5, 0.75 and 1. 

**Figure 6:**
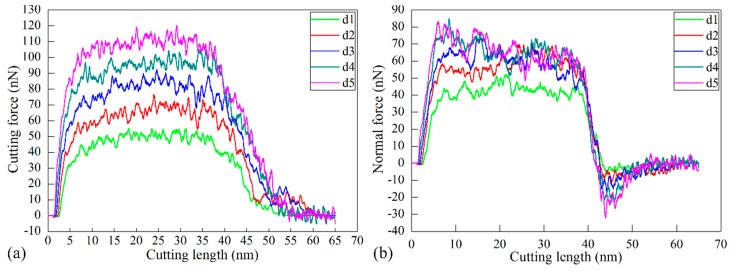
Variations of (**a**) cutting force and (**b**) normal force with cutting length during cutting processes with different DOCs.

Figure 6a,b plot the variation of cutting force and normal force with cutting length in each cutting process. It is can be seen from Figure 6 that the variation of the machining force for each DOC can be categorized into three zones, which is similar with the machining force for the DOC of 0.2 nm as described in the Section 3.1. In the first zone that corresponds to a cutting length of 0–10 nm. The material undergoes elastic deformation accompanied with a rapid increase of the machining force. In the second zone, which corresponds to a cutting length of 10–35 nm, the machining force remains stable with fluctuations around constant values after elastic–plastic transition, indicating that the cutting process is stable. In the third zone, which corresponds to a cutting length of 35–65 nm, the machining force decreases rapidly due to the separation of the cutting tool from the workpiece.

Figure 6 also suggests that the DOC has a significant influence on machining force variations. Figure 6a shows that in the first and second zones, the value of the cutting force is monotonously higher for a higher DOC. In the cutting length ranging from 10 to 35 nm, the average values of both cutting force and normal force increase with increasing *R*. Furthermore, the ratio of cutting force to normal force also increases with increasing *R*.

**Figure 7:**
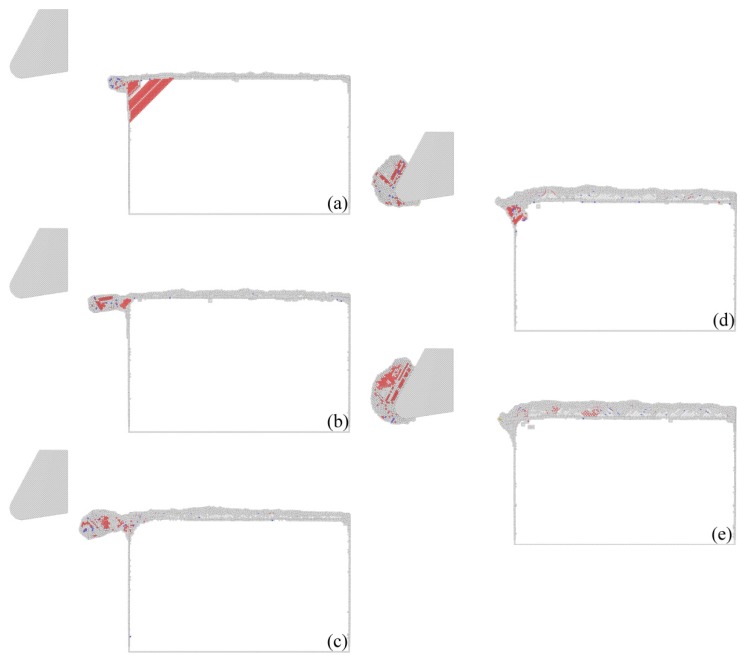
Cross-sectional views of instantaneous defect structures in machined workpiece materials with different DOCs. DOC: (**a**) d1; (**b**) d2; (**c**) d3; (**d**) d4; (**e**) d5. Atoms are colored to their CNA values.

Figure 7 presents cross-sectional views of instantaneous defect structures within materials after cutting processes with different DOCs. It can be found that there is no chip formed for any DOC that is no larger than d3. Furthermore, dislocation density within the workpiece material decreases with the increase of DOC. In contrast, there is a chip formed for d4 or d5. Furthermore, there are considerable dislocations in the formed chip. However, there is rather limited dislocation beneath the machined surface, which demonstrates that the formation of the chip lowers the dislocation density within the workpiece material. Figure 7 indicates that there is a transition of material removal mechanisms from plowing to cutting with the increase of DOC. The observed DOC-dependent material removal mode is in agreement with previous studies [21,22,23]. 

Figure 7 shows that when the DOC is 0.2 nm, there is a rather limited defect formed within the workpiece material when the cutting process is stable. However, when the cutting tool is very close to separating from the workpiece material, a slip plan that is inclined to the adjacent left free surfaces is activated due to strong stress concentration built in the intersections of the free surfaces. Although there is no chip formed for the DOC of 0.2 nm, there is little machining debris formed in the left side of the workpiece. When the DOC is 0.6 or 1 nm, there are considerable dislocations generated in the workpiece material, but the dislocation density is less than that for the DOC of 0.2 nm. Furthermore, there is still no chip formed, although the extent of the machining debris is increased. There are chips formed for the DOC of 1.5 or 2 nm, at which there are also dislocations on both sides of the groove. Furthermore, the chip volume and dislocation density increase with the increase of DOC.

**Figure 8:**
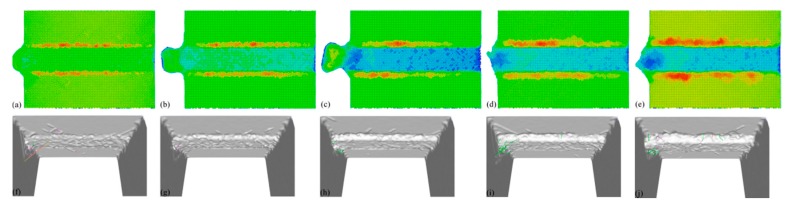
Machined surface morphologies (top row) and dislocation characterization (bottom row) in machined workpieces with different DOCs. DOC: (**a,f**) d1; (**b,g**) d2; (**c,h**) d3; (**d,i**) d4; (**e,j**) d5.

Figure 8 presents machined surface morphologies for different DOCs. While the material removal for DOCs no higher than d3 is dominated by plowing, Figure 6a–c show that displaced material is mainly accumulated on both sides of the formed groove, and cutting debris is also observed in the end of the workpiece. It is found that both the volume of surface pile up and the volume of debris are larger for higher DOCs. For DOCs higher than d3, there is also considerable surface pile up formed on both sides of the groove, although materials are mainly removed in the form of chip. Figure 8 also presents characterized dislocation structures within the workpiece material with different DOCs. It can be found that when the DOC is no larger than d3, dislocation mainly resides on the end of workpiece, and dislocation density is less than that at d4 or d5.

When the DOC is smaller than the cutting edge radius, the effective rake angle involved in the cutting process is always negative, which means that the compression stress applied by the cutting tool leads to extrusion of the displaced workpiece in the form of plowing, which consequently results in non-uniform material removal. With the increase of DOC, the effective negative rake angle is also increased, thus leading to decreased compress stress and increased shear stress. Consequently, uniform material removal can be achieved in the form of cutting, which leads to uniform machined surface quality. Upon further increase of DOC, however, the machined surface quality deteriorates due to serious plastic deformation in the material. The critical DOC for the transition of material removal mode from plowing to cutting at microscopic scale can be calculated analytically based on the geometry of the cutting tool. For a spherical tool with cutting edge radius (*r*) sliding on a flat substrate at a DOC (*d*), the critical depth (*d_c_*) for transition is given as [24]:

*d_c_* = *r*{1 − cos[tan^−1^(μ)]} (1)

where μ is the coefficient of friction at the sliding interface. For a μ in the range of 0–1, the derived critical depth *d_c_* is in good agreement with the simulation results.

**Figure 9:**
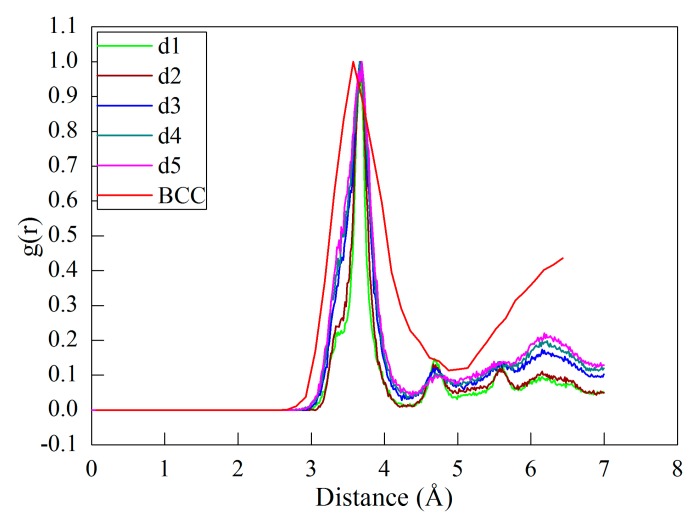
RDF analysis of phase transformation in the diamond cutting of cerium with different DOCs.

In addition to dislocation slip-dominated plasticity, the influence of DOC on the propensity of phase transformation in the diamond cutting process of cerium is also evaluated by performing RDF analysis on the workpiece. Figure 9 plots the RDF of the machined workpiece at different DOCs, and the RDF of the δ-Ce is also provided for reference. It is seen from Figure 9 that each DOC yields a similar RDF curve of the workpiece material. Furthermore, the first peak of the RDF curve of the machined surface with each DOC closely coincides with that of the BCC cerium, indicating that there is phase transformation from γ-Ce to δ-Ce in the machined surface. In particular for d4 and d5, corresponding to the cutting mode of material removal, there is the same phase transformation from γ-Ce to δ-Ce occurred in the formed chip. Figure 9 also indicates that the DOC slightly influences the propensity for phase transformation. With the increase of DOC, the intensity of the RDF peaks increases, indicating that the propensity for phase transformation from γ-Ce to δ-Ce increases.

### 3.3. Diamond Cutting Experiments

**Figure 10:**
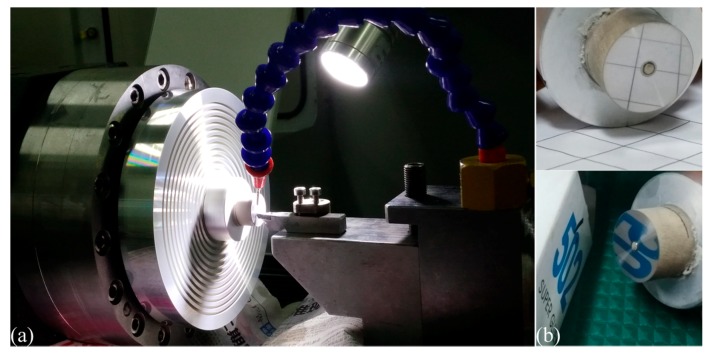
(**a**) Configuration of diamond cutting of cerium; (**b**) machined surface of cerium at the DOC of 5 μm.

Diamond cutting experiments of cerium with different DOCs are also carried out on a home-made NanoForm ultra-precision diamond turning lathe. The detailed configuration of this diamond cutting experiment is shown in Figure 10a. The 99.5% purity cerium workpiece has a cylinder shape with a diameter of 20 mm. The diamond cutting tool has a nose radius of 1 mm and a cutting edge radius of 60 nm. The diamond cutting tool has a rake angle of 0° and a clearance angle of 9°. The utilized spindle speed is 1000 rpm and the feed rate is 1 μm/rev. Prior to the formal cutting, a pre-cutting with a DOC of 10 μm is conducted to remove the oxide layer on the cerium workpiece. To address the influence of DOC on the diamond cutting of the cerium workpiece, four DOCs of 2, 5, 10 and 15 μm are considered. For each DOC, the machined surface quality of the cerium workpiece is characterized by a surface profiler and an atomic force microscope (AFM).

Figure 10b presents the machined surface obtained with the DOC of 5 μm, which shows that a mirror surface with considerable reflectance is achieved on the cerium workpiece. Table 1 lists the machined surface quality in terms of surface roughness (*Ra*) for the four DOCs, which indicates that a good surface quality can be achieved at a DOC of 5 or 10 μm. The surface quality for the smallest DOC of 2 μm is not good as the *Ra* is 133.2 nm. With an increase of DOC to 5 μm, the Ra is reduced to 46.2 nm, indicating that the surface quality is significantly improved. The surface quality is further refined at a DOC of 10 μm, at which the *Ra* is 37.6 nm. With a further increase of DOC to 15 μm, however, the machined surface quality deteriorates as the *Ra* is 64.1 nm. Table 1 demonstrates that the DOC-dependent surface roughness measured by the AFM is generally consistent with that obtained by the surface profiler. Table 1 also shows that the measured results by the AFM are smaller than those of the surface profiler for each DOC, due to a smaller measurement area.

**Figure 11:**
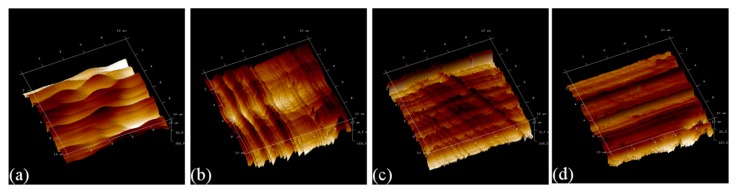
AFM image of machined surface morphology obtained with different DOCs. DOC of (**a**) 2μm; (**b**) 5 μm; (**c**) 10 μm and (**d**) 15 μm.

Figure 11 presents AFM images of the four machined surface obtained with different DOCs. It is found that for the DOC of 2 μm, the resulting surface is not uniform due to non-continuous material removal. Consequently, the value of surface roughness of the machined surface is high. With the increase of DOC, the propensity of continuous material removal is increased, thus a uniform machined surface quality with a decreased value of surface roughness is achieved. However, at the largest DOC, there are obvious tool marks remaining on the machined surface, and the resulting value of surface roughness is also high. It is known that plowing leads to non-continuous material removal, and cutting is desirable for achieving a high quality of machined surface due to continuous material removal in terms of chip formation [21]. Therefore, Figure 11 indicates that there is a transition of dominant material removal from plowing to cutting with the increase of DOC, which qualitatively agrees well with the results of MD simulations.

## 4. Summary

In summary, we performed MD simulations of diamond cutting of single crystalline cerium to evaluate the effect of DOC on the cutting process. It is found that the microscopic deformation of cerium workpieces under diamond cutting is dominated by dislocation nucleation and subsequent slip. In addition, there is phase transformation from γ-Ce to δ-Ce within both the machined surface and the formed chip. The sharpness of the cutting edge has a strong influence on the material removal and machined surface quality. While an atomically sharp cutting edge leads to uniform material removal in the form of chip, there is no chip formed for a blunt cutting edge due to the dominant plowing effect. Simulation results indicate that with the increase of DOC, there is a transition of material removal mode from plowing to cutting, which leads to increased machined surface quality. However, a further increase of DOC deteriorates machined surface quality due to serious plastic deformation. Diamond cutting experiments also show that the DOC has a strong influence on the material removal and relating machined surface quality, which qualitatively agree well with MD simulations.

## Figures and Tables

**Table 1:** Surface roughness *Ra* of machined surface of cerium.

Surface Roughness	d1	d2	d3	d4
*Ra* (Profiler, nm)	133.2	46.2	37.6	64.1
*Ra* (AFM, nm)	43.1	27.0	33.6	38.2
